# Translation, Cultural Adaptation, and Validation of the Greek Version of the 4 ‘A’s Test for Delirium Screening in Elderly Patients with Hip Fracture

**DOI:** 10.3390/clinpract16030058

**Published:** 2026-03-09

**Authors:** Maria Spyraki, Evanthia Dimitriou, Panagiotis Antzoulas, Georgios Karpetas, Francesk Mulita, Vasileios Leivaditis, Ejona Shaska, John Lakoumentas, Diamanto Aretha, Andreas Panagopoulos

**Affiliations:** 1 Department of Anesthesiology, General University Hospital of Patras, 26504 Patras, Greece; spyrakim@ac.upatras.gr (M.S.); drevadim@gmail.com (E.D.); giokar8@gmail.com (G.K.); adaretha@yahoo.gr (D.A.); 2 Department of Orthopedics, General University Hospital of Patras, 26504 Patras, Greece; p.antzoulas@hotmail.com (P.A.); andpan21@gmail.com (A.P.); 3 Department of Surgery, General Hospital of Aigio, 25100 Aigio, Greece; 4 Department of Cardiothoracic and Vascular Surgery, Westpfalz Klinikum, 67655 Kaiserslautern, Germany; vnleivaditis@gmail.com; 5 Department of Psychiatry, “Ali Mihali” Psychiatric Hospital, 9401 Vlora, Albania; zilja.jona@yahoo.it; 6 Department of Medical Physics, General University Hospital of Patras, 26504 Patras, Greece; john.lakoo@gmail.com

**Keywords:** delirium, hip fracture, 4AT, functional detection tool, elderly patients

## Abstract

**Background:** Delirium is a frequent and serious complication in elderly patients with hip fractures and is associated with adverse outcomes. Early identification requires a brief and reliable screening tool suitable for routine clinical practice. The 4 ‘A’s Test (4AT) is a rapid instrument for delirium detection that requires minimal training. **Objective:** To translate, culturally adapt, and validate the Greek version of the 4AT in elderly patients with hip fractures. Methods: A total of 103 patients aged ≥65 years who were admitted with hip fracture were enrolled. The 4AT was translated using a forward–backward translation process and culturally adapted according to established guidelines. Delirium diagnosis was established using DSM-5 criteria by trained clinicians, serving as the reference standard. The 4AT was administered independently within 3 h. Diagnostic accuracy was assessed by calculating sensitivity, specificity, positive predictive value (PPV), negative predictive value (NPV), and area under the receiver operating characteristic curve (AUC). The optimal cut-off was determined using Youden’s index. **Results:** At a cut-off score ≥4, the Greek 4AT demonstrated a sensitivity of 87.5% and specificity of 91.1%, with PPV 75% and NPV 96%. The AUC was 0.94, indicating excellent diagnostic performance. **Conclusions:** The Greek version of the 4AT is a valid and reliable screening tool for detecting delirium in elderly patients with hip fractures.

## 1. Introduction

Delirium is an acute and fluctuating neuropsychiatric syndrome characterized by disturbances in attention and awareness [1]. Clinical manifestations vary and may include disorientation, hallucinations, agitation, and inappropriate behavior in the hyperactive subtype, whereas hypoactive delirium is more often associated with lethargy and reduced responsiveness [2]. Its pathogenesis is multifactorial, involving an interaction between patient-related vulnerabilities, healthcare-related factors, and medication exposure [1].

Predisposing factors include advanced age, pre-existing cognitive impairment, sensory deficits, multimorbidity, and functional dependence. Superimposed precipitating factors—such as trauma, surgery, anesthesia, infections, dehydration, and certain medications including anticholinergics and antipsychotics—substantially increase the likelihood of delirium development [3,4,5].

Delirium affects up to 50% of hospitalized older adults and is strongly associated with adverse outcomes [6], including prolonged hospitalization, long-term cognitive decline, increased risk of dementia, and higher mortality [7]. Patients who develop delirium have nearly a threefold higher risk of death during hospitalization and within six months after discharge compared to those without delirium [8,9,10].

In emergency departments, delirium is present in approximately 7–20% of older adults [2,11], and prevalence may reach 89% among individuals with pre-existing cognitive impairment or dementia [12]. In surgical populations, particularly in post-anesthesia care units, reported incidence ranges from 3% to 54%, depending on the diagnostic criteria and timing of assessment [13].

Despite its clinical importance, delirium frequently remains underdiagnosed, with up to 83% of cases unrecognized [14]. This underdetection is partly attributable to the fluctuating nature of symptoms and partly to the limited implementation of sensitive and validated screening tools across different clinical settings, often resulting in reliance on subjective clinical judgment [15].

International recommendations, including those from Australia’s Commission on Safety and Quality in Health Care and the United Kingdom’s National Institute for Health and Care Excellence, advise routine delirium screening in hospitalized patients aged 65 years and older [15]. Although the Diagnostic and Statistical Manual of Mental Disorders, Fifth Edition (DSM-5), represents the reference standard for diagnosis [16], its application requires specialized training and is not easily integrated into routine clinical workflows.

Several brief instruments have therefore been developed to facilitate early detection. These include the 4 ‘A’s Test (4AT), the Confusion Assessment Method (CAM) and its intensive care adaptation (CAM-ICU), the Brief Confusion Assessment Method (bCAM), the 3-Minute Diagnostic CAM (3D-CAM), the Single Question in Delirium (SQiD), and the Nursing Delirium Screening Scale (Nu-DESC) [17,18]. Their principal advantage lies in their rapid administration, making them particularly suitable for perioperative use [19,20]. In high-risk populations such as elderly patients with hip fractures, prompt recognition and management of delirium constitute key indicators of quality hospital care [21].

To date, no validation study of a delirium screening instrument has been conducted specifically in Greek hip fracture patients. The 4AT is a brief (<2 min), user-friendly tool designed for routine practice. It evaluates four core features of delirium: alertness, orientation (AMT4), attention, and acute change or fluctuating course. Previous studies have demonstrated high sensitivity and specificity of the 4AT in hospitalized older populations, including patients with hip fractures [22].

The present study aimed to translate and culturally adapt the 4AT into Greek and to evaluate its diagnostic accuracy for detecting delirium in elderly patients undergoing hip fracture surgery in an in-hospital setting. Sensitivity was defined as the proportion of correctly identified delirium-positive cases, and specificity as the proportion of correctly identified delirium-negative cases.

## 2. Materials and Methods

### 2.1. Study Design

This diagnostic accuracy study was conducted at the University Hospital of Patras, Greece, between April 2022 and September 2023 to translate, culturally adapt, and validate the Greek version of the 4 ‘A’s Test (4AT). This diagnostic accuracy study was designed and reported in accordance with the Standards for Reporting Diagnostic Accuracy Studies (STARD) statement [23]. The completed STARD checklist is provided in the Supplementary Materials (Table S1). Where applicable, elements of the Strengthening the Reporting of Observational Studies in Epidemiology (STROBE) guidelines were also considered to ensure transparent reporting.

The methodological process comprised two sequential phases. First, the 4AT underwent forward and backward translation, followed by evaluation of content validity to ensure cultural and conceptual equivalence, in accordance with the International Society for Pharmacoeconomics and Outcomes Research (ISPOR) guidelines for translation and cross-cultural adaptation [24]. Second, the diagnostic accuracy of the Greek 4AT was assessed by comparison with the Diagnostic and Statistical Manual of Mental Disorders, Fifth Edition (DSM-5) criteria, which served as the reference standard for delirium diagnosis.

The study protocol was approved by the Ethics Committee on Human Experimentation and the Scientific Board of the General University Hospital of Patras, Greece (Approval No. UOP17, 28 January 2022). All procedures were conducted in accordance with the ethical principles of the Declaration of Helsinki. Written informed consent was obtained from all participants or, when applicable, from their legally authorized representatives [25]. The study also adhered to the Guidelines for Reporting Reliability and Agreement Studies (GRRAS).

### 2.2. Translation and Cultural Adaptation

Forward Translation: Following formal authorization from the developers of the 4AT, two independent bilingual translators with experience in medical terminology translated the instrument from English into Greek. The two forward translations were compared and synthesized into a single consensus version after discussion between the translators. This preliminary Greek version was then reviewed by the research team to ensure semantic accuracy, conceptual equivalence, and suitability for use in the intended clinical context.

Backward Translation: The reconciled Greek version was subsequently translated back into English by a professional native English-speaking translator who was blinded to the original instrument. The back-translated version was systematically compared with the original English 4AT to identify potential inconsistencies or conceptual deviations. Any discrepancies were discussed and resolved to achieve linguistic and conceptual alignment between versions.

Cognitive Debriefing and Pilot Testing: Content validity was further assessed by a panel of six healthcare professionals (physicians and nurses) experienced in the management of delirium. Their feedback focused on clarity, comprehensibility, and clinical applicability. A pilot test involving 15 patients was then conducted to evaluate understandability and cultural appropriateness in real-world conditions. Based on the results of the pilot phase, minor refinements were made, followed by final proofreading and confirmation of the definitive Greek version.

### 2.3. Participants and Recruitment

**Figure 1:**
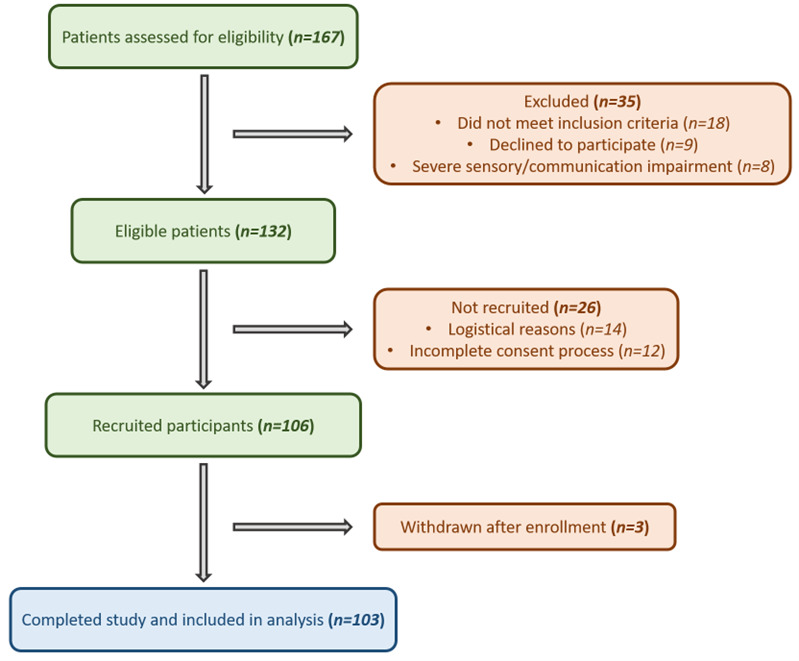
Flow diagram of participant selection. Flowchart illustrating the number of patients assessed for eligibility, excluded (with reasons), recruited, and finally included in the analysis.

The population of the study included patients aged ≥ 65 years old who underwent non- elective surgery due to hip fracture. The age threshold of ≥65 years was selected in accordance with international delirium screening recommendations and previous 4AT validation studies, which define this age group as representing a high-risk geriatric population. Informed consent was taken by two experienced and well-trained anaesthesiologists in the field of delirium. Patients who met the exclusion criteria shown in Table 1 were not eligible to participate. A flow diagram summarizing patient screening, exclusions, recruitment, and final inclusion in the analysis is presented in Figure 1.

### 2.4. Statistical Analysis

Only participants with complete data on gender, age, 4AT score, and delirium classification according to DSM-5 criteria (“YES” or “NO”) were included in the statistical analysis. Normality of continuous variables was assessed using the Shapiro–Wilk test. As distributions deviated from normality, continuous variables are presented as median with interquartile range (Q1–Q3), whereas categorical variables are reported as absolute frequencies and percentages.

Comparisons between delirium groups were performed using the Wilcoxon rank-sum test for continuous variables and Pearson’s chi-squared test for categorical variables. All statistical tests were two-sided, and statistical significance was defined at the 5% level.

To evaluate the independent association between clinical variables and delirium status, multivariable logistic regression analysis was conducted, modeling delirium classification as the dependent variable and age, gender, and 4AT score as predictors.

Diagnostic performance of the 4AT was assessed through receiver operating characteristic (ROC) curve analysis. The optimal cut-off point was determined using Youden’s index, and corresponding sensitivity, specificity, positive predictive value (PPV), and negative predictive value (NPV) were calculated.

All statistical analyses and graphical visualizations were performed using R statistical software (version 4.1.2) with RStudio (“Ghost Orchid” release). Both programs are open-source and distributed under the GNU General Public License.

### 2.5. Assessments

All eligible participants underwent both index testing (4AT) and reference standard assessment (DSM-5 criteria) at the bedside within a 30-min interval, in order to minimize the impact of the fluctuating nature of delirium.

Two pairs of experienced assessors independently performed the evaluations: one pair administered the 4AT, while the other applied the DSM-5 diagnostic criteria. The assessors were blinded to each other’s findings throughout the assessment process to prevent diagnostic bias. Following completion of both evaluations and subsequent unblinding of the results, the assessment procedure for each participant was finalized.

### 2.6. AT Assessment

**Figure 2:**
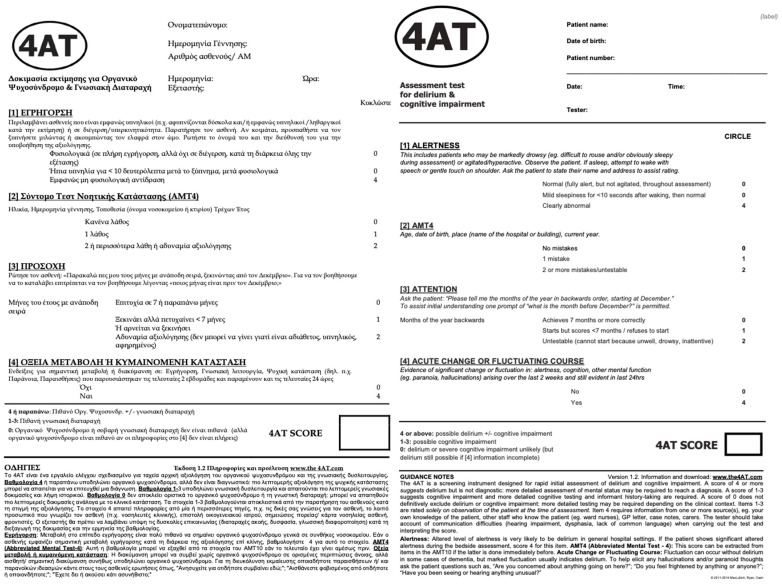
The 4AT assessment tool. English version retrieved from https://www.the4at.com/ accessed on 7th of October 2025.

The 4AT was administered by trained clinical researchers with experience in delirium assessment. The instrument comprises four domains: (1) alertness, (2) AMT4 (abbreviated mental test—four items), (3) attention, and (4) acute change or fluctuating course (Figure 2).

The total score ranges from 0 to 12. A score of ≥4 suggests possible delirium, with or without underlying cognitive impairment. Scores between 1 and 3 indicate potential cognitive impairment, while a score of 0 makes delirium or severe cognitive impairment unlikely, provided that information regarding acute change or fluctuation is complete.

For the purposes of diagnostic accuracy analysis in this study, a cut-off value of ≥4 was used to classify participants as delirium-positive, whereas scores of 0–3 were considered delirium-negative.

Administration time of the 4AT did not exceed 5 min per participant.

### 2.7. Reference Standard Assessment

The reference standard diagnosis of delirium was established according to the criteria of the Diagnostic and Statistical Manual of Mental Disorders, Fifth Edition (DSM-5) [16].

Assessment was conducted by two trained clinical researchers with experience in delirium evaluation. The evaluators performed structured clinical assessments following the acquisition of informed consent. DSM-5 criteria were applied systematically to determine the presence or absence of delirium. Additional methodological documentation is available in the Supplementary Materials (Figure S1).

## 3. Results

A total of 167 patients were screened for eligibility, of whom 132 met the inclusion criteria (Figure 1; STARD flow diagram). Among these, 106 were recruited, and 103 completed the study and were included in the final analysis.

The median age of the cohort was 82 years (IQR 75–87), and 67 participants (65.1%) were female. According to DSM-5 criteria, delirium was diagnosed in 24 patients (23.3%).

### 3.1. Univariate and Multivariable Analyses

Univariate analysis demonstrated no significant association between gender and delirium status (*p* = 0.578). In contrast, age was significantly associated with delirium (*p* < 0.001). Patients with delirium had a higher median age compared with those without delirium (87 [84–91.5] vs. 78 [73–85] years).

The overall median 4AT score was 1 (IQR 0–4). When stratified by delirium status, the median 4AT score was 0 (IQR 0–1) in the non-delirium group and 6 (IQR 4–8) in the delirium group (*p* < 0.001).

The 4AT score showed a moderate positive correlation with age (Spearman’s ρ = 0.507, *p* < 0.001).

To assess independent predictors of delirium, multivariable logistic regression analysis was performed including age, gender, and 4AT score. In the adjusted model, age did not remain statistically significant (β = 0.112, SE = 0.062, *p* = 0.072), whereas the 4AT score retained strong independent predictive value (β = 0.671, SE = 0.158, *p* < 0.001). Gender was not significantly associated with delirium in either univariate or multivariable analysis.

Table 2 summarizes descriptive statistics, results of univariate testing, and multivariable logistic regression analysis.

### 3.2. Diagnostic Accuracy of the 4AT

Using Youden’s index to determine the optimal threshold for predicting delirium, a cut-off score of ≥4 was identified. At this threshold, the 4AT demonstrated:

- Sensitivity: 87.5%
- Specificity: 91.1%
- Positive predictive value (PPV): 75.0%
- Negative predictive value (NPV): 96.0%
- Area under the ROC curve (AUC): 0.946 (95% CI: 0.906–0.986)

**Figure 3:**
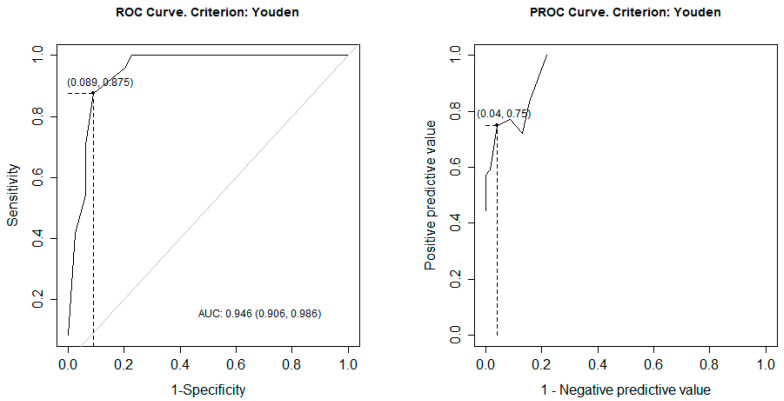
ROC and PROC curves. Legend ROC; receiver operating characteristic, PROC; predictive receiver operating characteristic, AUC; area under the curve.

These findings indicate excellent overall diagnostic discrimination. The ROC curve is presented in Figure 3.

### 3.3. Performance of Individual 4AT Components

Diagnostic metrics were also calculated for each of the four individual components of the 4AT (Table 3). Among them, item A4 (acute change or fluctuating course) demonstrated the highest discriminative performance (AUC 0.891; 95% CI: 0.811–0.971), contributing most substantially to the overall diagnostic accuracy.

Item A1 (alertness) showed high specificity (0.962) but low sensitivity (0.125), whereas items A2 (AMT4) and A3 (attention) demonstrated balanced sensitivity and moderate specificity. The composite 4AT score (SUM ≥4) achieved the highest overall AUC (0.946; 95% CI: 0.906–0.986).

Table 3 presents detailed sensitivity, specificity, predictive values, and ROC AUC estimates for each component.

## 4. Discussion

Early and accurate recognition remains one of the central challenges in the diagnosis and management of delirium. Despite increasing awareness and guideline recommendations, delirium continues to be substantially underdetected in routine clinical care, particularly in busy surgical and emergency environments [26,27]. Studies consistently demonstrate that systematic screening significantly improves detection rates compared with routine physician assessment alone [28,29]. The fluctuating nature of delirium, combined with time constraints and competing clinical priorities, contributes to missed diagnoses. Therefore, implementation of structured screening tools is increasingly regarded not merely as supportive but as essential to quality perioperative care. Within this framework, our findings demonstrate that the Greek version of the 4AT is a reliable, feasible, and diagnostically accurate instrument for detecting delirium in elderly patients undergoing hip fracture surgery. The high sensitivity and specificity observed in our cohort further support its practical utility in high-risk surgical populations.

Our results align closely with international validation studies conducted across diverse healthcare systems. The systematic review and meta-analysis by Tieges et al. reported pooled sensitivity and specificity values of 88% for the 4AT [30], findings remarkably consistent with our sensitivity (87.5%). Comparable performance has been documented in emergency departments [17,31], acute geriatric wards, postoperative units, and mixed medical populations [32,33,34]. The original validation study by Bellelli et al. reported sensitivity approaching 90% in hospitalized older adults [35], while Shenkin et al. demonstrated robust diagnostic performance in a multicenter prospective comparison between 4AT and CAM [34]. These findings collectively indicate that the diagnostic stability of the 4AT persists across languages, healthcare systems, and clinical contexts. Our slightly higher specificity (91.1%) may reflect the relatively homogeneous nature of our study cohort, consisting exclusively of elderly hip fracture patients managed within a structured perioperative pathway.

The excellent discriminative capacity observed in our study (AUC 94.6%) further reinforces the clinical reliability of the Greek 4AT [36,37,38,39,40,41]. Similar AUC values have been reported in orthopedic and surgical populations [33,39], suggesting that the tool maintains strong predictive accuracy even in settings characterized by postoperative physiological fluctuations. Importantly, delirium in hip fracture patients is associated with increased postoperative complications, institutionalization, prolonged hospital stay, and excess mortality [34,41,42]. Large registry-based studies confirm that delirium independently predicts adverse outcomes even after adjustment for age and comorbidities [42,43]. Consequently, validated screening in this population carries substantial implications not only for detection but also for potential early intervention and outcome modification.

Beyond statistical significance, the magnitude of the observed differences underscores their clinical relevance. The marked separation in median 4AT scores between the non-delirium (0) and delirium (6) groups reflects a substantial divergence in cognitive status rather than a marginal statistical effect. Importantly, the cut-off value of ≥4 corresponds to the established threshold for probable delirium in prior validation studies and therefore represents a clinically meaningful boundary rather than an arbitrary statistical division. The high negative predictive value (96%) further supports the practical utility of the tool, indicating that a score below the threshold reliably excludes delirium in this high-risk population. Although age was statistically significant in univariate analysis, its loss of significance in multivariable modeling suggests that the 4AT score captures clinically relevant variation beyond chronological age alone. Thus, the statistically significant findings reported in this study are accompanied by effect sizes consistent with meaningful diagnostic discrimination in routine practice.

The presence of false-positive cases in patients with dementia or mild cognitive impairment deserves particular attention. Differentiating delirium superimposed on dementia from baseline cognitive impairment remains clinically challenging [34,44]. However, previous validation studies indicate that the 4AT retains acceptable specificity in cognitively impaired cohorts [31,32,35]. In our study, misclassification rates were low, and the overlap observed likely reflects the inherent diagnostic complexity rather than tool inadequacy. Moreover, early identification of cognitive vulnerability may still be clinically beneficial, as patients with dementia are at particularly high risk for delirium-related complications [44].

Examination of individual 4AT components provides further interpretative depth. Acute change or fluctuating course (A4) consistently emerges as one of the most discriminative elements of delirium [34,37,38]. Our findings confirm its strong specificity. In contrast, the alertness domain (A1) demonstrated lower sensitivity, consistent with previous observations [30,37,38]. Delirium-related alterations in arousal may be subtle, transient, or masked by postoperative sedation, potentially explaining this pattern. Additionally, the binary scoring format of A1 may reduce granularity in detecting mild abnormalities [39,40]. Conversely, structured attention tasks, such as the Months Backwards test, generally demonstrate high sensitivity but reduced specificity in frail elderly cohorts [31,35], likely due to baseline cognitive deficits unrelated to acute delirium. These patterns highlight the multidimensional structure of delirium assessment and reinforce the importance of evaluating the total score rather than isolated domains.

From an implementation perspective, the brevity and minimal training requirements of the 4AT offer significant advantages. Unlike CAM, which requires structured training and algorithmic interpretation, the 4AT can be administered rapidly without extensive preparation [34,45]. This feature is particularly relevant in orthopedic trauma settings, where rapid turnover and perioperative time pressures may hinder prolonged cognitive assessment. Emerging implementation studies suggest that integration of standardized screening tools into routine workflows improves documentation and may facilitate multidisciplinary management strategies [28,29,45]. Therefore, validation of the Greek version represents not only a methodological achievement but also a practical step toward structured delirium surveillance in Greek hospitals.

Methodologically, our study benefits from rigorous cross-cultural adaptation procedures, ensuring semantic fidelity and contextual appropriateness. The use of DSM-5 criteria as the reference standard remains appropriate, as DSM-5 continues to define the diagnostic framework for delirium [16]. Independent blinded assessments further minimized observer bias. The delirium prevalence of 23.3% aligns with rates reported in multicenter hip fracture studies [42,43], supporting external validity.

Nonetheless, certain limitations warrant acknowledgment. Exclusion of patients with severe communication barriers may restrict generalizability to more heterogeneous clinical populations. Additionally, while the sample size was sufficient for validation purposes, larger multicenter studies would enhance statistical precision and allow subgroup analyses across age strata and comorbidity profiles. Future research may also explore whether systematic implementation of the Greek 4AT translates into measurable reductions in morbidity, length of stay, or mortality, outcomes increasingly emphasized in perioperative geriatric care research [43,45]. It should also be emphasized that this study is observational in design. Although statistically significant associations were identified—particularly in relation to age and 4AT score—these findings should not be interpreted as causal relationships. The regression analyses were conducted to explore associations between clinical variables and delirium status rather than to establish causality. As with all observational studies, residual confounding by unmeasured variables (e.g., comorbidity burden, perioperative medication use, baseline frailty) cannot be excluded. Therefore, the reported associations should be interpreted within the context of diagnostic validation rather than causal inference.

## 5. Limitations

This study has several limitations. First, the study was conducted in a single tertiary hospital, which may limit the generalizability of the findings to other clinical settings. Second, although the overall sample size was adequate for diagnostic validation purposes, the number of delirium-positive cases was relatively limited, which may have restricted the ability to perform detailed subgroup analyses, including age-stratified comparisons. Third, age was analyzed as a continuous variable, and age-specific subgroup analyses were not performed. Future multicenter studies with larger cohorts could further explore potential variations in delirium prevalence and screening performance across different age groups. Moreover, the inclusion and exclusion criteria resulted in a relatively well-defined cohort of elderly patients undergoing hip fracture surgery. While this homogeneity enhances internal validity and reduces clinical heterogeneity, it may limit generalizability to broader patient populations, such as younger surgical patients, individuals with severe communication barriers, or those managed in non-surgical settings. However, hip fracture patients represent one of the highest-risk groups for delirium in routine clinical practice, and the demographic characteristics and delirium prevalence observed in our study are consistent with previously reported real-world data. Therefore, although extrapolation to all hospitalized populations should be undertaken cautiously, the cohort remains representative of the target high-risk orthopedic population commonly encountered in acute care hospitals. Finally, patients with severe dysphasia, sensory impairment, or inability to communicate in Greek were excluded, which may limit applicability to more heterogeneous clinical populations.

## 6. Conclusions

This study demonstrates that the Greek version of the 4 ‘A’s Test (4AT) is a valid, reliable, and clinically applicable screening instrument for the rapid detection of delirium in elderly patients with hip fractures. When compared with DSM-5 criteria, the tool showed excellent diagnostic accuracy, supporting its suitability for use in high-risk perioperative settings. Owing to its brevity, ease of administration, and minimal training requirements, the 4AT can be readily integrated into routine clinical workflows across acute hospital environments. Systematic screening with a standardized instrument such as the 4AT may enhance early recognition of delirium, facilitate timely intervention, and potentially improve patient outcomes.

Although the single-center design and exclusion of patients with severe communication or sensory impairment may limit generalizability, the findings provide robust support for the implementation of the Greek 4AT in clinical practice. Further multicenter studies involving larger and more diverse populations are warranted to confirm these results and to evaluate the impact of structured delirium screening on healthcare outcomes. Overall, the Greek 4AT represents a practical and effective tool that can strengthen perioperative delirium detection and contribute to improved quality of care for older patients undergoing hip fracture surgery.

## Figures and Tables

**Table 1:** Inclusion and exclusion criteria.

**Inclusion criteria**
Aged 65 years or over
Hip fracture surgery
**Exclusion criteria**
Acute life-threatening illness requiring time-critical intervention or transmission to the ICU
Coma
Severe dysphasia
Severe and/or combined visual and hearing impairment
Unable to communicate in Greek
Refuse to participate

Legend* *ICU; intensive care unit.

**Table 2:** Descriptive statistics.

Variable	Cohort [*N* = 103]	Hypothesis Testing Procedure			Logistic Regression Modeling		
		Delirium “NO” [*N* = 79]	Delirium “YES” [*N* = 24]	*p*-Value	Beta Estimate	STD. Error	*p*-Value
GENDER F	67 (65.05%)	53 (67.09%)	14 (58.33%)	-	-	-	-
GENDER M	36 (34.95%)	26 (32.91%)	10 (41.67%)	0.587	−0.225	0.766	0.769
AGE	82 (75–87)	78 (73–85)	87 (84–91.5)	**<0.001**	0.112	0.062	0.072
4AT SCORE	1 (0–4)	0 (0–1)	6 (4–8)	**<0.001**	0.671	0.158	**<0.001**

Legend STD; standard, F; female, M; male.

**Table 3:** Metrics of individual pillars of 4AT.

4AT PILLAR	CUTOFF	SENS.	SPEC.	PPV	NPV	ROC AUC (95% CI)
A1	≥4	0.125	0.962	0.500	0.784	0.544 (0.473–0.614)
A2	≥1	0.667	0.810	0.516	0.889	0.770 (0.659–0.880)
A3	≥1	0.833	0.646	0.417	0.927	0.765 (0.663–0.867)
A4	≥4	0.833	0.949	0.833	0.949	0.891 (0.811–0.971)
SUM	≥4	0.875	0.991	0.750	0.960	0.946 (0.906–0.986)

Legend* *SENS; sensitivity, SPEC; specificity, PPV; positive predictive value, NPV; negative predictive value, ROC AUC; receiver operating characteristic area under the curve, CI; confidence interval, A1; first item (alertness), A2; second item (abbreviated mental test), A3; third item (attention), A4; fourth item (acute change or fluctuating course), SUM; summation.

## Data Availability

The original contributions presented in this study are included in the article/Supplementary Material. Further inquiries can be directed to the corresponding author.

## Supplementary Materials

The following supporting information can be downloaded at: https://www.mdpi.com/article/10.3390/clinpract16030058/s1, Figure S1: STARD 2015 checklist for reporting diagnostic accuracy studies; Table S1: STARD 2015 Checklist.
