# Supplementary material for: Translation, Cultural Adaptation, and Validation of the Greek Version of the 4 ‘A’s Test for Delirium Screening in Elderly Patients with Hip Fracture
Source: Clin Pract. 2026 Mar 9;16(3):58. doi: 10.3390/clinpract16030058 (PMC13025903; doi:10.3390/clinpract16030058)
Supplement: Supplementary file 1 [file clinpract-16-00058-s001.zip › clinpract-4138086-supplementary.pdf]

Supplementary File S1

Table S1. STARD 2015 Checklist

Section & Topic	Item	Reported in Manuscript
TITLE OR ABSTRACT	1	Identified as a diagnostic accuracy study (Title; Abstract; Section 2.1)
ABSTRACT	2	Structured abstract including Background, Objective, Methods, Results, Conclusions
INTRODUCTION	3	Scientific and clinical background (Section 1)
INTRODUCTION	4	Study objectives and hypotheses (End of Section 1)
METHODS – Study Design	5	Prospective diagnostic accuracy design (Section 2.1)
Participants	6	Eligibility criteria (Section 2.3)
Participants	7	Identification of eligible participants (Section 2.3)
Participants	8	Setting, location, and dates (Sections 2.1 and 2.3)
Participants	9	Consecutive sampling of eligible patients (Section 2.3)
Test Methods	10a	Description of index test (Section 2.6; Figure 2)
Test Methods	10b	Description of reference standard (Section 2.7)
Test Methods	11	Rationale for reference standard (Section 2.7; Discussion)
Test Methods	12a	Definition of 4AT cut-off (Sections 2.6 and 2.4)
Test Methods	12b	Definition of reference standard (DSM-5; Section 2.7)
Test Methods	13a	Blinding of index test assessors (Section 2.5)
Test Methods	13b	Blinding of reference standard assessors (Section 2.5)
Analysis	14	Statistical methods for diagnostic accuracy (Section 2.4)
Analysis	15	Handling of indeterminate results (None reported)
Analysis	16	Handling of missing data (Complete-case analysis; Section 2.4)

Analysis	17	Analysis of variability (ROC analysis; Section 2.4; Discussion)
Analysis	18	Sample size and justification (Section 2.3; Discussion limitations)
RESULTS – Participants	19	Flow of participants (Figure 1 – STARD flow diagram)
RESULTS – Participants	20	Baseline demographic and clinical characteristics (Results; Table 2)
RESULTS – Participants	21a	Distribution of disease severity (Results; Table 2)
RESULTS – Participants	21b	Distribution of alternative diagnoses (Discussion)
RESULTS – Participants	22	Time interval between index and reference tests (Section 2.5)
Test Results	23	Cross-tabulation of index test vs reference standard (Table 2; ROC analysis)
Test Results	24	Diagnostic accuracy estimates (Results; Section 2.4)
Test Results	25	Adverse events (None observed)
DISCUSSION	26	Study limitations and generalizability (Discussion section)
DISCUSSION	27	Implications for practice (Discussion; Conclusion)
OTHER INFORMATION	28	Registration (Not applicable – diagnostic validation study)
OTHER INFORMATION	29	Protocol availability (Available upon request)
OTHER INFORMATION	30	Funding and role of funders (Funding section)

Figure S1. STARD 2015 checklist for reporting diagnostic accuracy studies.

Section & Topic	No	Item
**TITLE OR ABSTRACT**		
	**1**	Identification as a study of diagnostic accuracy using at least one measure of accuracy (such as sensitivity, specificity, predictive values, or AUC)
**ABSTRACT**		
	**2**	Structured summary of study design, methods, results, and conclusions (for specific guidance, see STARD for Abstracts)
**INTRODUCTION**		
	**3**	Scientific and clinical background, including the intended use and clinical role of the index test
	**4**	Study objectives and hypotheses
**METHODS**		
Study design	**5**	Whether data collection was planned before the index test and reference standard were performed (prospective study) or after (retrospective study)
Participants	**6**	Eligibility criteria
	**7**	On what basis potentially eligible participants were identified (such as symptoms, results from previous tests, inclusion in registry)
	**8**	Where and when potentially eligible participants were identified (setting, location and dates)
	**9**	Whether participants formed a consecutive, random or convenience series
Test methods	**10a**	Index test, in sufficient detail to allow replication
	**10b**	Reference standard, in sufficient detail to allow replication
	**11**	Rationale for choosing the reference standard (if alternatives exist)
	**12a**	Definition of and rationale for test positivity cut-offs or result categories of the index test, distinguishing pre-specified from exploratory
	**12b**	Definition of and rationale for test positivity cut-offs or result categories of the reference standard, distinguishing pre-specified from exploratory
	**13a**	Whether clinical information and reference standard results were available to the performers/readers of the index test
	**13b**	Whether clinical information and index test results were available to the assessors of the reference standard
Analysis	**14**	Methods for estimating or comparing measures of diagnostic accuracy
	**15**	How indeterminate index test or reference standard results were handled
	**16**	How missing data on the index test and reference standard were handled
	**17**	Any analyses of variability in diagnostic accuracy, distinguishing pre-specified from exploratory
	**18**	Intended sample size and how it was determined
**RESULTS**		
Participants	**19**	Flow of participants, using a diagram
	**20**	Baseline demographic and clinical characteristics of participants
	**21a**	Distribution of severity of disease in those with the target condition
	**21b**	Distribution of alternative diagnoses in those without the target condition
	**22**	Time interval and any clinical interventions between index test and reference standard
Test results	**23**	Cross tabulation of the index test results (or their distribution) by the results of the reference standard
	**24**	Estimates of diagnostic accuracy and their precision (such as 95% confidence intervals)
	**25**	Any adverse events from performing the index test or the reference standard
**DISCUSSION**		
	**26**	Study limitations, including sources of potential bias, statistical uncertainty, and generalisability
	**27**	Implications for practice, including the intended use and clinical role of the index test
**OTHER INFORMATION**		
	**28**	Registration number and name of registry
	**29**	Where the full study protocol can be accessed
	**30**	Sources of funding and other support; role of funders

Completed checklist indicating where each reporting item recommended by the Standards for Reporting Diagnostic Accuracy Studies (STARD 2015) is addressed within the manuscript to ensure transparent and comprehensive reporting.
